# Lateral hypothalamic kindling induces manic-like behavior in rats: a novel animal model

**DOI:** 10.1186/s40345-014-0007-8

**Published:** 2014-06-14

**Authors:** Osama A Abulseoud, Ulas M Camsari, Christina L Ruby, Khalid Mohamed, Noha M Abdel Gawad, Aimen Kasasbeh, Mehmet Y Yüksel, Doo-Sup Choi

**Affiliations:** Department of Psychiatry and Psychology, Mayo Clinic, 200 First Street SW, Rochester, MN 55905 USA; Department of Molecular Pharmacology and Experimental Therapeutics, Mayo Clinic, 200 First Street SW, Rochester, MN 55905 USA; Department of Neurosurgery, Mayo Clinic, 200 First Street SW, Rochester, MN 55905 USA

**Keywords:** Lateral hypothalamus, Kindling, Manic, Circadian genes, Lithium, Valproic acid

## Abstract

The lateral hypothalamus integrates critical physiological functions such as the sleep-wake cycle, energy expenditure, and sexual behaviors. These functions are severely dysregulated during mania. In this study, we successfully induced manic-like behavioral phenotypes in adult, male Wistar rats through bilateral lateral hypothalamic area kindling (LHK). To test the validity of the model, we studied the effect of standard antimanic medications lithium (47.5 mg/kg) or valproic acid (200 mg/kg) twice/day for 15 days in attenuating manic-like behaviors in the LHK rat. Compared with pre-kindling behaviors, LHK rats displayed significantly increased sexual self-stimulation (*P* = 0.034), excessive rearing (*P* = 0.0005), feeding (*P* = 0.013), and grooming (*P* = 0.007) during the kindling interval. LHK rats also drank more alcohol during the mania-induction days compared with baseline ethanol consumption levels (*P* = 0.01). Moreover, LHK rat exhibited increased total locomotor activity (*P* = 0.02) with reduced rest interval (*P* < 0.001) during the mania induction and post-mania days compared with baseline activity levels and rest intervals. Chronic administration of lithium or valproic acid significantly attenuated manic-like behaviors in the LHK rat model. Given the behavioral phenotype and the response to standard antimanic medications, the LHK rats may provide a model for studying manic psychopathology in humans.

**Electronic supplementary material:**

The online version of this article (doi:10.1186/s40345-014-0007-8) contains supplementary material, which is available to authorized users.

## Background

Bipolar disorder is a common, recurrent medical illness (Merikangas et al*.*^2007^) characterized by at least one manic episode (type I) or alternating episodes of mania (or hypomania in type II) and depression (American Psychiatric Association 2000; Goodwin and Jamison 2007; Judd et al*.*^2002^). In the United States, up to 1% of the general population will suffer from bipolar disorder type I (Merikangas et al*.*^2007^) during their lifetime, leading to significant disability (Murray and Lopez 1997) and high rates of completed suicide (Inskip et al. 1998; Tondo et al. 2003). The acute manic episode, the hallmark of bipolar disorder, is a medical emergency that frequently requires hospitalization (Hirschfeld et al*.*^2006^). Current pharmacological interventions can be divided into three groups: lithium, anticonvulsants, and atypical antipsychotics. The efficacy of these agents as monotherapies in the treatment of acute mania in randomized controlled trials approximates 50% response and only 25% remission (McElroy and Keck 2000; Perlis et al*.*^2006^). The suboptimal efficacy of antimanic medications stems, at least in part, from the fact that the underlying neural mechanisms triggering and maintaining a manic episode remain largely unknown.

Animal models simulating the manic illness are essential to understanding the complex pathophysiology of this disorder. There are currently two major categories of animal models that exhibit manic-like behaviors. In the first, the manic-like behavior is induced by behavioral stress (e.g., sleep deprivation) or chemical treatment (e.g., methamphetamine). In the second, specific gene or pathway alterations such as circadian locomotor output cycles kaput (CLOCK); or extracellular signal regulated protein kinases 1 (ERK1) known to be implicated in bipolar disorder are introduced into animals (Chen et al*.*^2010^). Despite being highly valuable in studying this complex disorder, current animal models have several limitations. For example, in the models with genetic alterations, although the proteins encoded by the genes of human and rodents are highly homologous, the regulation of gene transcription by transcription factors or epigenetics, or the regulation of gene translation by microRNAs, can vary extensively between humans and rodents. Furthermore, the gene-behavior relationships of some behaviors may be conserved only to a certain extent between the human and animal genomes (Chen et al. 2010). Similarly, amphetamine treatment in rodents is a well-accepted mania model, while in humans, it could induce a syndrome virtually indistinguishable from paranoid schizophrenia. These observations highlight the significant need to identify new valid mania models that exhibit more phenotypic features of the illness and link certain aspects of manic-like behaviors to the underlying neural networks. In this study, we propose a novel animal model through kindling of the lateral hypothalamic area in rats.

The hypothalamus coordinates various neural systems that mediate different functional responses (Bouret and Simerly 2004), integrates the motivational aspects of behavior with visceral-motor responses, regulates energy homeostasis (Williams et al*.*^2001^), initiates sexual behavior (Beauregard et al*.*^2001^; Ferretti et al*.*^2005^; Hamann et al. 2004; Redoute et al. 2000), and synchronizes the rhythms of all biological processes with environmental changes to achieve the optimal adaptive response (Mendlewicz and Linkowski 1987). The lateral hypothalamus in particular has been implicated in the processing of sensory information and the expression of behaviors associated with hunger and thirst, aggression, and reproduction (Bernardis and Bellinger, 1993). The lateral hypothalamus is involved in mediating general arousal and sensory sensitization associated with motivational behavior (de Lecea et al. 2012). Several lines of evidence suggest the involvement of the hypothalamus in the pathophysiology of mania. Structural brain imaging studies suggest a dilation of the third ventricle in the region where hypothalamic nuclei are located adjacent to its walls, indirectly implicating reduced hypothalamic volume (Bhadoria et al. 2003; Cousins et al*.*^2010^; Pearlson et al*.*^1997^; Strakowski et al. 1993). Furthermore, two postmortem studies (Bielau et al. 2005; Brisch et al. 2011) showed significant reduction in the volume of the hypothalamic region in patients with bipolar disorder type I compared to those with major depression and healthy controls.

Moreover, preclinical studies in rats (Kruk 1991), pigs (Ettrup *et al.*^2012^), and monkeys (Lacan et al*.*^2008^) have shown that high-frequency stimulation of the hypothalamus engages functional circuits involved in different behaviors reminiscent of mania such as hypersexuality, aggression, increased locomotor activity, and disturbed sleep-wake cycle. In the female Gottingen pig, transient aggressive territorial behavior followed by increased locomotor activity and no sleep for the first night was reported following bilateral stimulation of the lateral hypothalamus (Ettrup et al. 2012). In the vervet monkey, bilateral high-frequency stimulation of the ventromedial hypothalamus elicited a transient period of agitated and sexual behavior (Lacan et al. 2008). Based on the aforementioned evidence, we hypothesized that the lateral hypothalamus was the logical target to induce manic-like behaviors. However, there remains the question of the stimulation pattern optimal for induction of manic behavior.

Kindling is a well-established concept referring to the development of a full seizure as a result of the delivery of repeated subthreshold stimuli. Amygdala kindling has been proposed by Post and Weiss (1989) as a model of epilepsy to explain the efficacy of the anticonvulsant carbamazepine in treatment of mania in patients with bipolar disorder. However, the symptomatology induced by amygdala kindling was distinct from any caused by mania (Post and Weiss 1989). In this study, we investigated a novel animal model of mania induced by delivering brief stimulation pulses to the lateral hypothalamus.

## Methods

### Animals

The experiments were done on adult male Wistar rats (age 12 to 16 weeks, weight 250 to 300 gm at beginning of experiment) obtained from Charles River Laboratories International, Inc. (Wilmington, MA, USA). Rats were housed in individual cages on a 12-h light/dark cycle (lights on at 6 a.m.), free supply of food (*ad libitum*), and tap water. All experimental procedures were approved by the Mayo Clinic Institutional Animal Care and Use Committee.

### Experimental groups

Phenotype experiments had four groups (*n* = 8 each) designed to compare the effect of kindling vs. sham at the lateral hypothalamus and two other control brain regions: the nucleus accumbens (NAC) shell and the infra-limbic cortex (ILC). The NAC shell was chosen for its role in reward circuitry (Russo and Nestler 2013) which is known to be intimately involved in manic behaviors. The IL cortex is a subregion of the prefrontal cortex (PFC). In the rat, the PFC consists of medial prefrontal cortex (mPFC) and orbitofrontal cortex (OFC). The mPFC is functionally linked to the limbic system (Vertes 2006) and anatomically is subdivided into infralimbic (IL), prelimbic (PL), and dorsal anterior cingulate (dAC) (Czeh et al. 2008). The PL cortex, homologues to dorsolateral prefrontal cortex in humans (Laroche et al. 2000) projects to insular cortex, thalamus, amygdala, hippocampus and ventral tegmental area (Vertes 2006). The PL cortex is thought to be directly involved in limbic and cognitive function (Marquis et al. 2007). The IL cortex in rats, on the other hand, is analogues to the subgenual Brodmann area 25 in humans (Drevets et al*.*^1997^; Ongur et al*.*^2003^). Deep brain stimulation of area 25 is associated with successful treatment of refractory depression (Mayberg et al. 1999, 2005).

**Figure 1:**
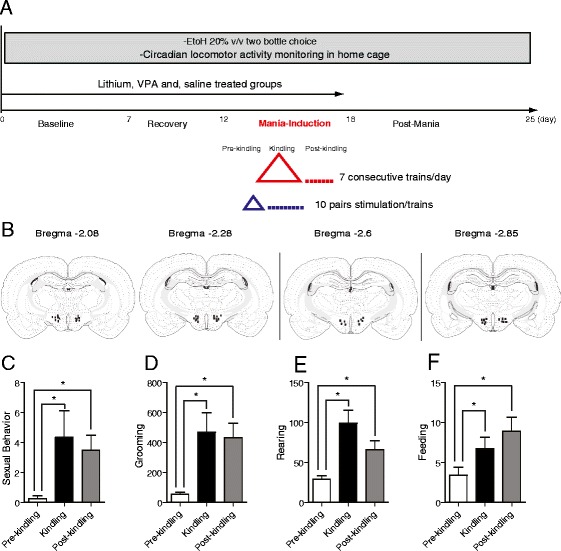
**Study design, electrode location, and kindling-induced manic-like behaviors.** Study design shown in **(A)** included the baseline phase for 7 days, followed by surgical implantation of stimulating electrode and recovery for 5 days before mania induction took place for 5 days. Each day, the animal was allowed an initial pre-kindling habituation period for 30 min followed by kindling phase consisting of seven consecutive trains of escalating volts (1 through 7 V) with 2-min rest interval between trains. Each train consisted of 10 pairs of stimulation (10 s) alternating with rest (30 s). The animal remained in the monitored cage for 30 min during the post-kindling period before it was returned back to home cage for 7 days (post-mania) before euthanasia and brain collection was performed. Locomotor activity counts and voluntary ethanol consumption were monitored continuously throughout the study period. Animals were assigned to lithium, valproate (VPA), or saline for 15 days. **(B)** An illustration of stimulating electrode tip locations plotted within the dorso-medial part of the LHA regions. AP coordinates were between −1.7 and −2.8 from bregma according to Paxinos Atlas. Manic-like behaviors were elicited in LHK rats. Significant increase in the frequency of sexual behavior **(C)**, duration of grooming **(D)**, the frequency of rearing **(E)**, and feeding behaviors **(F)** were evident during the kindling process and persisted during the post-kindling interval by two-way ANOVA; *P < 0.05 by *post hoc* test; *n* = 10 to 12 per group. Data is expressed as mean ± SEM.

Predictive validity experiments had three groups (*n* = 12 each). All received lateral hypothalamic area kindling (LHK) and were assigned to lithium, VPA, or saline treatment to test the efficacy of standard antimanic medications in attenuating manic-like behavior. Animals were allowed a 1-week habituation interval before surgery and mania induction procedures followed by 7 days of post-mania before euthanasia and brain collection were performed. A diagrammatic illustration of the study design is depicted in Figure 1A.

### Surgical implantation of stimulating electrodes

Anesthetized animals (isoflurane inhalation, 3.0% during induction and 1% to 1.5% during maintenance) were secured in the stereotaxic apparatus (David Kopf Instruments, Tujunga, CA, USA) and the skull leveled between bregma and lambda. Bipolar stimulating electrodes (#MS303 twisted stainless steel, outer diameter 125 μm, Plastics One, Inc., Roanoke, VA, USA) were implanted bilaterally into the lateral hypothalamic area (A/P, −2.28; M/L, ±2.7; D/V, −8.5 mm from skull surface) and in the nucleus accumbens shell in another group of animals (A/P, +1.2; M/L, ±2.7; D/V, −8.5 mm from skull surface) and in the ILC in a third group (A/P, +3.24; M/L, ±2.4; D/V, −5.1 mm from skull surface). All electrodes were implanted with a 20° angle to allow enough room to attach the stimulating cords in both sides. Electrodes were secured to the skull using dental cement and three screws. Animals were closely observed over 5 days post-surgery. Animals with any neurological signs of brain damage were excluded from the study.

### Mania-induction procedure

All procedures took place in the early light phase between 6 a.m. and 8 a.m. during mania induction days. The animal was placed individually into a clear Plexiglas cage (12″ × 12″ × 30″, with ordinary bedding and food pellets on the floor) for 30 min at the beginning of the experiment to habituate to the new environment. This interval was called the pre-kindling interval and was followed by 60 min of kindling using the following stimulation parameters: bipolar configuration, 180 Hz/s frequency, 500 μs pulse width, and 10-s pulse durations followed by 30 s of rest. Seven trains were applied, each consisting of 10 pairings of 10-s duration stimulation pulses alternating with 30 s of rest, and 2 min of rest were allowed between trains. Stimulation amplitude was started at 1 V and was increased by 1 V for each stimulation train. These stimulation parameters were chosen empirically to simulate the clinical DBS parameters used in the voltage-dependent mania case report (Chopra et al. 2011). Following all seven kindling trains, the animal was kept in the observation chamber for 30 more minutes as post-kindling interval. Subsequently, the stimulating cord was disconnected and the animal was returned back to the home cage. The exact same procedure was repeated for five consecutive days. The number of mania-induction days was also chosen arbitrary to replicate a manic episode in humans that usually lasts several days.

### Monitoring and quantifying behaviors during the process of mania induction

Continuous video recording of behaviors was performed over the 2-h mania-induction session. Recorded video files were transferred and stored in two separate external hard drives. Each animal recording was reviewed independently and behaviors were coded separately by two trained blinded raters (UC and MY), and target behaviors were reviewed by the principal investigator of the study (OA). The four primary behaviors were coded: (1) sexual behavior, the number of any oral contact with the genital area (erected or flaccid penis or testicles) or immediate perigenital area (Additional file 1: Video S1); (2) rearing behavior, the number of episodes of standing on the two hind limbs for more than 5 s duration (Additional file 2: Video S2); (3) grooming behavior, the total time (in 5-second increments) of repeated mouthing of any body part except the genital and perigenital areas or repeated movement of the fore or hind paws over the snout, face, head, trunk, or tail (Additional file 3: Video S3); and (4) feeding behavior, the number of times where the animal is observed holding and chewing food pellets. Chewing without food or chewing bedding was not considered feeding behavior (Additional file 4: Video S4). We developed this novel method of continuous monitoring, identifying, and quantifying clear individual manic-like behaviors reminiscent of clinical mania rating scales.

### 24-h sleep-wake cycle and locomotor activity monitoring in home cage during baseline, mania-induction, and post-mania days

Circadian locomotor activity counts in home cage were recorded using an infrared motion detector interfaced with a computerized data acquisition system (ClockLab, ActiMetrics, Wilmette, IL, USA) and later analyzed using MATLAB (The MathWorks, Inc., Natick, MA, USA). Total locomotor activity counts (bout analysis) and total activity time for light and dark phases were measured separately for each day of the experiment during baseline, mania-induction days, and post-mania days. The non-activity time was calculated by subtracting total activity time (minutes) from 720 min (12-h light/dark phase). This outcome measure was used as a surrogate marker for rest or sleep.

### Voluntary ethanol consumption via two-bottle choice paradigm

Animals in both the phenotype experiments and predictive validity experiments were offered 20% (*v*/*v*) ethanol vs. tap water in a two-bottle choice paradigm throughout the experiment. Water, ethanol, and food consumption were measured by weighing the animal, the two bottles, and the food pellets in the feeding tray daily. To avoid the confounding of side preference, we switched the bottle side every time fluid measurements were taken.

To examine the potential confounding effect of ethanol on the induced behaviors, we compared manic-like behaviors between voluntary ethanol-drinking and water-only drinking rats (*n* = 8 each).

### Lithium and valproate sodium treatment

Lithium (47.5 mg/kg; Sigma, St. Louis, MO, USA) or VPA (200 mg/kg; Sigma, St. Louis, MO, USA) and saline (1 ml) IP twice/day were administered for 15 consecutive days (10 days before and 5 days during mania-induction). No lithium, VPA, or saline were administered during the post-mania days. Lithium carbonate was dissolved in distilled water 30 mg/mL, and VPA was dissolved in 0.9% 50 mg/mL saline according to manufacturer guidelines. The chronic treatment and the doses of VPA and lithium were based on previous studies (Castro et al*.*^2009^; Feier et al. 2013; Frey et al*.*2006a, b) demonstrating the safety and yielding therapeutic lithium blood levels in the range of 0.8 to 1.1 mEq/L, closely resembling therapeutic levels used to treat humans with bipolar disorder (Nolen and Weisler 2013).

### Euthanasia and brain histology for verification of electrode tip location

Animals were lightly anesthetized in a CO_2_ chamber and euthanized by rapid decapitation around the same time (2000 hours for all animals). The brain was carefully collected and fixed in paraformaldehyde solution for 24 h followed by 30% sucrose solution for 1 week then covered with optimal cooling temperature (OCT) compound for cryostat sectioning (Ted Pella Inc., Redding, CA, USA) and stored at −80°C until histology was performed. Brains were sectioned on a cryostat (50 μm) and stained with hematoxylin and eosin. Verification of electrode tip location was done according to the atlas of Paxinos and Watson (1998).

### Statistical analysis

The behavioral outcomes for each day during the three observation intervals (i.e., pre-kindling, kindling, and post-kindling) were first analyzed with repeated measures analyses of variance (ANOVAs). For simplicity, the sum of all events of an individual parameter that took place during all days in the observation interval was then re-analyzed. To test for differences in individual behavioral manifestations, two-way ANOVAs were used with factors of time (baseline vs. mania induction vs. post-mania) and groups (LHK-Sham vs. LHK-stim vs. NAC, vs. ILC) or factors of time (baseline vs. mania induction vs. post-mania) and treatment (LHK/saline vs. LHK/VPA vs. LHK/lithium). When a significant interaction was found, a *post hoc* testing was performed to determine pairwise differences. One-way ANOVAs were used to examine differences in individual behaviors during kindling between groups (LHK vs. NAC vs. ILC) and to compare behaviors during each interval (pre-kindling, kindling and post-kindling) within the LHK group. All data were presented as means ± SEM. Results were considered significant when *P* < 0.05.

## Results

A total of 84 animals were used in this study. Data from nine subjects were excluded for animals that died (*n* = 3), that pulled the electrode prior to or during kindling (*n* = 2), or in cases where electrodes were found outside the target brain region (*n* = 4) as confirmed by histological examination. To assess for potential electrode-associated brain tissue damage, we have examined the motor function of each rat post-implantation for any neurological deficit. Furthermore, we examined brain tissue histology for any evidence of hemorrhage or infarction. A diagram for the projected electrode tip location in the LHK group is shown in Figure 1B.

### LHK induces manic-like behavior in naïve rats

Sexual behavior was observed exclusively in animals that underwent LHK (Additional file 5: Figure S1A). Within the LHK group, the frequency of sexual behavior showed a significant increase during kindling and post-kindling compared with pre-kindling levels: one-way ANOVA *F*_2,33_ = 3.364, *P* = 0.0468, *post hoc t* test pre-kindling vs. kindling (*t* = 2.416, *df* = 11, *P* = 0.034), and pre-kindling vs. post-kindling (*t* = 3.493, *df* = 11, *P* = 0.005, Figure 1C). A significant increase in the total time spent in grooming behavior was evident in the LHK compared with the kindled NAC or kindled ILC groups during kindling (*F*_2,21_ = 5.821, *P* = 0.009, Additional file 5: Figure S1B). Also in the LHK group, the duration of grooming behavior showed a significant increase during kindling and post-kindling compared with pre-kindling levels: one-way ANOVA *F*_2,33_ = 5.76, *P* = 0.007, *post hoc t* test pre-kindling vs. kindling (*t* = 3.268, *df* = 11, *P* = 0.007), pre-kindling vs. post-kindling (*t* = 4.029, *df* = 11, *P* = 0.002, Figure 1D). A significant increase in the frequency of rearing behavior was observed in the LHK compared with the kindled NAC and ILC groups during kindling (*F*_2,21_ = 13.78, *P* = 0.0001, Additional file 5: Figure S1C). In the LHK group, the frequency of rearing behavior showed a significant increase during kindling and post-kindling compared with pre-kindling levels: one-way ANOVA *F*_2,33_ = 8.799, *P* = 0.0009, *post hoc t* test pre-kindling vs. kindling (*t* = 4.88, *df* = 11, *P* = 0.0005), pre-kindling vs. post-kindling (*t* = 3.391, *df* = 11, *P* = 0.006, Figure 1E). A significant increase in feeding behavior was seen in the LHK group compared with the kindled NAC and ILC groups during kindling (*F*_2,23_ = 12.67, *P* = 0.0002, Additional file 5: Figure S1D). In the LHK group, the frequency of feeding behavior showed a significant increase during kindling and post-kindling compared with pre-kindling levels: one-way ANOVA *F*_2,33_ = 3.838, *P* = 0.031, *post hoc t* test pre-kindling vs. kindling (*t* = 2.932, *df* = 11, *P* = 0.013), pre-kindling vs. post-kindling (*t* = 2.956, *df* = 11, *P* = 0.013, Figure 1F).

To examine the potential confounding effect of concomitant ethanol consumption on elicited manic-like behaviors or the possibility that differences in behaviors could have resulted from differential ethanol intake between the groups, we compared manic-like behaviors between two LHK groups, water only and ethanol (20% *v*/*v* versus water) drinking groups (*n* = 8 each). We did not observe any significant differences in induced manic-like behaviors between the two groups (Additional file 6: Figure S2).

### LHK-induced manic-like behaviors are attenuated by standard antimanic medications VPA and lithium

**Figure 2:**
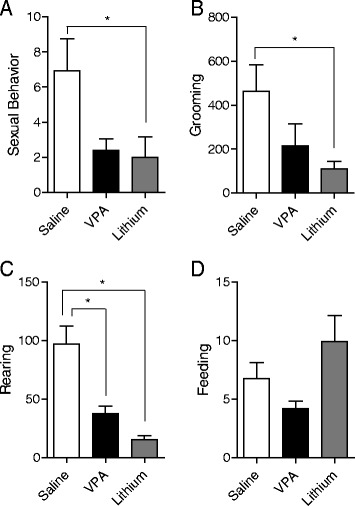
**The effects of lithium and VPA on sexual, grooming, rearing, and feeding behaviors.** This figure shows that lithium, but not VPA, significantly attenuated the frequency of **(A)** sexual behavior (*P = 0.024) and **(B)** the duration of grooming behavior (*P = 0.027). Both lithium and VPA reduced the frequency of rearing behavior [*P < 0.0001] **(C)**, but neither lithium nor VPA had an effect on the frequency of feeding behavior (*P = 0.065) **(D)** by two-way ANOVA; *P < 0.05 by *post hoc* test; *n* = 10 to 12 per group. Data is expressed as mean ± SEM.

Lithium, but not VPA, significantly attenuated the frequency of sexual behavior (*F*_2,31_ = 4.189, *P* = 0.024, Figure 2A) and the duration of grooming behavior (*F*_2,34_ = 4.02, *P* = 0.027, Figure 2B). Both lithium and VPA reduced the frequency of rearing behavior (*F*_2,34_ = 18.3, *P* < 0.0001, Figure 2C), but neither lithium nor VPA had an effect on the frequency of feeding behavior (*F*_2,31_ = 2.978, *P* = 0.065, Figure 2D).

### LHK is associated with reward-seeking behavior in the form of significant increase in voluntary ethanol consumption that is not attenuated by lithium or VPA treatment

Two-way ANOVA with time (baseline, mania-induction, and post-mania) and group (LHT sham, LHK, NAC, ILC) factors showed significant effect for time (*F*_2,46_ = 11.39, *P* < 0.0001), and group (*F*_2,46_ = 8.64, *P* = 0.0007) and an interaction between the two factors (*F*_4,46_ = 3.22, *P* = 0.026). Subsequent *post hoc* testing revealed a significant increase in ethanol consumption during mania-induction compared with the baseline interval in the LHK (*t* = 4.707, *P* < 0.01) and in the NAC group (*t* = 3.071, *P* < 0.05) but not in the ILC group (*t* = 2.79, *P* > 0.05).

**Figure 3:**
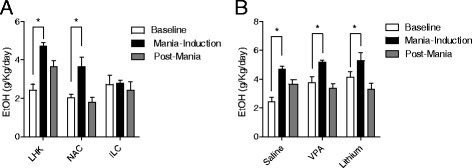
**Effect of Kindling on ethanol consumption.** Depicts the increased ethanol consumption in kindled LH, NAC, and ILC rats during mania-induction compared to baseline intervals (*P = 0.026) **(A)**. The significant increase in ethanol consumption remained during the post-mania interval only in the LHK group (baseline vs. post-mania LHK (*t* = 2.66, *P* < 0.05), NAC (*t* = 0.44, *P* > 0.05), and ILC (*t* = 0.536, *P* > 0.05). Neither lithium nor VPA attenuated LHK-induced increase in ethanol consumption (*P* = 0.12) **(B)** by two-way ANOVA; *P < 0.05 by *post hoc* test; *n* = 10 to 12 per group. Data is expressed as mean ± SEM.

The significant increase in ethanol consumption remained during the post-kindling interval only in the LHK group, baseline vs. post-mania LHK *t* = 2.66, *P* < 0.05; NAC *t* = 0.44, *P* > 0.05; and ILC *t* = 0.536, *P* > 0.05, Figure 3A. Neither lithium nor VPA treatment had a significant effect on ethanol consumption during mania induction by two-way ANOVA, (*F*_4,58_ = 1.86, *P* = 0.12, Figure 3B).

Of note, neither lithium nor VPA had a significant effect on weight gain, (Additional file 7: Figure S3A) or food consumption (Additional file 7: Figure S3B). However, lithium-treated rats consumed significantly more water (Additional file 7: Figure S3C) compared to saline or VPA-treated.

### LHK is associated with significant increase in total activity counts and reduced rest intervals during both light and dark phases

**Figure 4:**
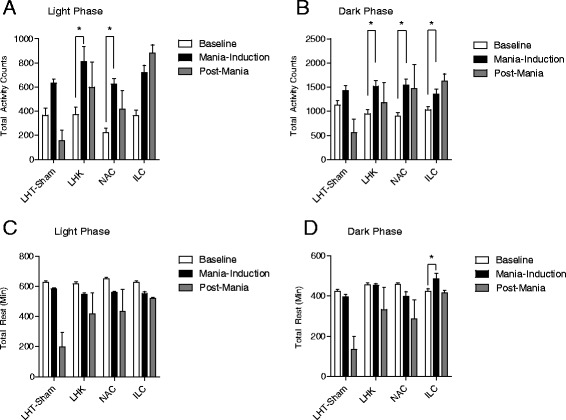
**The impact of mania induction on total locomotor activity and rest intervals during light and dark phases.** A significant increase in the total activity counts during the light phase **(A)** during mania induction compared with the baseline interval. Interaction (*P* < 0.006) in the LHK (*P < 0.001), and NAC (*P < 0.01), but not in the LHT-sham (*P* > 0.05), or ILC groups (*P* > 0.05). Similarly, increased activity during the dark phase **(B)** was evident. Interaction (*P* = 0.02) in the LHK (*P < 0.001), NAC (*P < 0.05), and ILC (*P < 0.05), but not in the LHT-sham (*P* > 0.05). Total rest interval during light phase **(C)** shows a significant decrease with interaction (*P* = 0.009) during mania induction compared with the baseline interval in all groups, the LHK (*P < 0.001), NAC (*P < 0.001), ILC (*P < 0.01), and in the LHT-sham (*P < 0.05). Total rest interval during dark phase **(D)** shows no significant decrease in total duration of rest during mania induction compared with the baseline interval in the LHK (*P* > 0.05), NAC (*P* > 0.05), or LHT-sham (*P* > 0.05) but and a significant increase in the ILC (*P* < 0.05) with an interaction (*P* = 0.0005) by two-way ANOVA; *P < 0.05 by *post hoc* test; *n* = 8 to 12 per group. Data is expressed as mean ± SEM.

A significant increase in the total activity counts during the light phase was evident between groups. An interaction between time (baseline, mania-induction, and post-mania) and group (LHK-sham, LHK, NAC, and ILC; *F*_6,32_ = 3.68, *P* < 0.006) and subsequent *post hoc* testing revealed a significant increase in activity counts during mania-induction days compared with the baseline interval in the LHK (*t* = 7.52, *P* < 0.001), and NAC (*t* = 3.907, *P* < 0.01), but not in the LHT-sham (*t* = 2.46, *P* > 0.05), or ILC groups (*t* = 2.64, *P* > 0.05, Figure 4A).

Examining the total activity counts during the dark phase where the animals are normally active using two-way ANOVA shows also significant difference between groups. An interaction between time and group (*F*_6,32_ = 2.97, *P* = 0.02) and subsequent *post hoc* testing revealed a significant increase in activity counts during mania-induction compared with the baseline interval in the LHK (*t* = 4.73, *P* < 0.001), NAC (*t* = 2.77, *P* < 0.05), and ILC (*t* = 2.66, *P* < 0.05) but not in the LHT-sham (*t* = 1.17, *P* > 0.05, Figure 4B).

Total rest interval during light phase shows a significant difference between groups. An interaction between time and group (*F*_6,32_ = 2.97, *P* = 0.009) and subsequent *post hoc* testing revealed a significant decrease in total duration of rest during mania-induction compared with the baseline interval in all groups, the LHK (*t* = 6.764, *P* < 0.001), NAC (*t* = 5.626, *P* < 0.001), ILC (*t* = 3.323, *P* < 0.01), and in the LHT-sham (*t* = 3.173, *P* < 0.05, Figure 4C).

Total rest interval during dark phase shows a significant difference between groups. An interaction between time and group (*F*_6,32_ = 2.97, *P* = 0.0005) and subsequent *post hoc* testing revealed no significant decrease in total duration of rest during mania-induction compared with the baseline interval in the LHK (*t* = 1.04, *P* > 0.05), NAC (*t* = 0.585, *P* > 0.05), or LHT-sham (*t* = 1.33, *P* > 0.05) and a significant increase in the ILC (*t* = 2.78, *P* < 0.05, Figure 4D).

### LHK-induced increased activity and reduced rest intervals are attenuated by standard antimanic medications VPA and lithium

**Figure 5:**
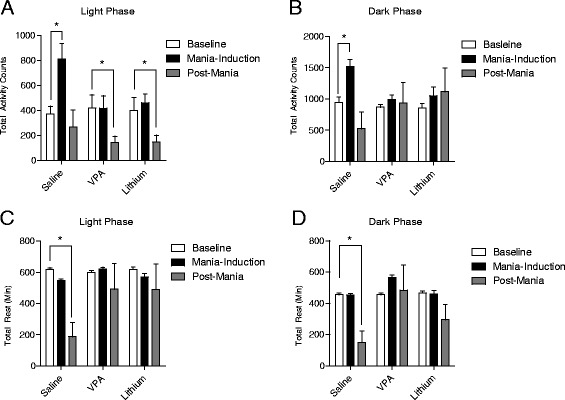
**Impact of 15-day treatment with saline, VPA and lithium.** On kindling-induced changes in total locomotor activity and rest intervals during light and dark phases. Comparing baseline with mania-induction during light phase **(A)** shows a significant increase in total activity counts only in saline (*P = 0.011), but not in VPA (*P* > 0.05), or lithium-treated (*P* > 0.05) groups with an interaction (*P = 0.01), while a significant reduction in activity counts during the post-kindling interval was seen in both VPA (*P = 0.038), and lithium-treated (*P = 0.019) animals but not in saline-treated rats (*P* > 0.05) by two-way ANOVA; *P < 0.05 by *post hoc* test; *n* = 10 to 12 per group. Data is expressed as mean ± SEM. Contrary to saline-treated rats, VPA and lithium prevented the kindling-induced increase in activity counts during dark phase **(B)**. Interaction (*P* = 0.0001), baseline vs. mania-induction in saline-treated group (*P < 0.001), VPA-treated (*P* > 0.05), and lithium-treated (*P* > 0.05) rats by two-way ANOVA; *P < 0.05 by *post hoc* test; *n* = 10 to 12 per group. Data is expressed as mean ± SEM. A significant reduction in rest interval observed during the post-kindling compared to pre-kindling days in both light **(C)** and dark **(D)** phases only in saline-treated rats (*P < 0.001), but not in the VPA (*P* > 0.05) or the lithium-treated (*P* > 0.05) groups. Comparing baseline vs. post-mania rest intervals during dark phase shows that saline-treated group had significant reduction (*P < 0.001) in rest interval that was abolished in both VPA (*P* > 0.05) and lithium-treated (*P* > 0.05) groups by two-way ANOVA; *P < 0.05 by *post hoc* test; *n* = 10 to 12 per group. Data is expressed as mean ± SEM.

Two-way ANOVA shows a significant interaction between time and treatment (*F*_4,54_ = 3.69, *P* = 0.01) and subsequent *post hoc* testing comparing baseline with mania-induction revealed a significant increase in total activity counts during light phase only in saline (*t* = 3.63, *P* = 0.011), but not in VPA (*t* = 1.36, *P* > 0.05), or lithium-treated (*t* = 0.495, *P* > 0.05) groups. During the post-mania interval, saline-treated animals returned back to baseline activity, baseline vs. post-mania saline-treated group (*t* = 0.092, *P* > 0.05), while the activity level decreased significantly in VPA (*t* = 2.33, *P* = 0.038), and lithium-treated (*t* = 4.55, *P* = 0.019) animals (Figure 5A).

Similar attenuating effect for lithium and VPA on dark phase activity counts during mania-induction days was also evident. A significant interaction between time and treatment (*F*_4,54_ = 3.69, *P* = 0.0001) was observed, and subsequent *post hoc* testing comparing baseline with mania-induction activity levels revealed a significant increase in total activity counts during dark phase only in the saline-treated group (*t* = 5.056, *P* < 0.001), but not in VPA-treated (*t* = 0.45, *P* > 0.05), or lithium-treated (*t* = 2.23, *P* > 0.05) groups (Figure 5B).

As expected, the increased activity was associated with reduced rest interval observed during the post-mania days in both light (*F*_4,54_ = 3.69, *P* = 0.0001) and dark (*F*_4,54_ = 3.69, *P* = 0.0001) phases only in saline-treated animals: light phase rest interval during baseline vs. post-mania intervals (*t* = 5.416, *P* < 0.001), whereas no significant differences were detected in the VPA-treated (*t* = 2.151, *P* > 0.05) or the lithium-treated (*t* = 0.85, *P* > 0.05) groups (Figure 5C) and also in comparing baseline vs. post-mania rest intervals during dark phase, saline-treated group show significant reduction (*t* = 8.918, *P* < 0.001) that was abolished in both VPA (*t* = 0.892, *P* > 0.05) and lithium-treated (*t* = 0.940, *P* > 0.05) groups (Figure 5D).

## Discussion

This study provides compelling evidence for the utility of the LHK rat as an appropriate model for multiple behavioral aspects of mania. Our findings demonstrate that the LHK rat presents a unique manic-like behavioral pattern including hypersexuality, increased grooming, rearing, and feeding behaviors. Moreover, mania induction increased voluntary ethanol consumption and locomotor activity while reducing rest for 7 days post-mania.

Sexual behavior in the form of genital self-stimulation was observed exclusively in the LHK rat during the kindling and post-kindling intervals (Figure 1C). Hypersexuality is an established criterion for diagnosing a manic episode in humans (American Psychiatric Association 2000). In this study, we adopted the frequency of genital licking as a marker for hypersexual behavior rather than the usual method of counting the frequency of male copulatory behaviors with receptive estrous females (Fiorino and Phillips 1999; Scotti et al*.*^2011^) to avoid the confounding element of potential stimulation of sexual behavior by the presence of a receptive female. Genital self-stimulation in male rats is well known to take place in the context of sexual behavior consistent with previous models (Roybal et al. 2007; Scotti et al. 2011); the frequency of sexual behavior in the LHK rat was reduced by lithium (Figure 2A), adding predictive validity to the model.

Interestingly, the excessive grooming behavior of non-genital areas was also elicited during the mania-induction process. Animals were observed to go into what seemed to be an ‘intense excitement’ with frequent rearing followed by excessive grooming and then exhibit erection and start genital licking. This pattern of development of manic-like behaviors was nearly uniform across subjects. The full pattern was occasionally seen, but the behavior did not always reach the genital self-stimulation phase. We have consistently observed the emergence of the same induced manic-like behaviors throughout all kindling days. However, induced manic-like behaviors tended to appear at progressively lower stimulation amplitudes

Important to the LHK model is the effect of specific antimanic pharmacotherapy. Lithium, but not VPA, significantly attenuated the duration of grooming behavior (Figure 2B), while the increase in rearing during LHK was attenuated by both lithium and VPA (Figure 2C). Attenuation of amphetamine-induced rearing is a well-established behavioral effect of therapeutically relevant lithium and VPA concentrations (Shaltiel et al*.*^2007^). Neither lithium nor VPA had a significant effect on the increased frequency of feeding behavior evident in the LHK rat (Figure 2D). Interestingly, studies in humans with comorbid bipolar and eating disorders show similar poor efficacy of standard antimanic medications in treating the eating component (McElroy et al. 2005). Similarly, the reward-seeking behavior in the form of increased voluntary ethanol consumption was significantly increased during mania-induction (Figure 3A) and was not attenuated by lithium or VPA treatment (Figure 3B). Clinical studies suggest that lithium treatment does not decrease alcohol intake in patients with bipolar disorder (Lejoyeux and Ades 1993). However, in the current study, we tested the efficacy of fixed dose monotherapy with lithium or VPA. It is possible that other dosing regimens or a combination of both medications could be more effective in reducing alcohol consumption than either one alone, as suggested in some studies (Kemp et al*.*^2009^; Salloum et al*.*^2005^). Further experiments are needed to explore these relevant issues.

A striking feature of our model is elevated hyperactivity during both the light and dark phases. Total activity counts during mania induction and the 7 days following reached twice as high as the baseline activity levels (Figure 4A,B). Combined with the significant reduction in total duration of sleep time (Figure 4C,D) and the ‘overexcitement’ picture observed during the kindling process, it suggests that these animals spend almost all their time ambulating. This fits well with clinical observations of a manic episode: pacing and reduced sleep. Both lithium and VPA reduced activity (Figure 5A,B) and increased rest intervals (Figures 5C,D) concomitant with the reduction of other manic-like behaviors, adding validity to the LHK model.

The drug response profile demonstrated by lithium and VPA in just attenuating but not completely abolishing manic-like behaviors resembles the efficacy of these medications in treating humans with mania (Greil et al. 1997; Hartong et al. 2003). Combining this with the results of the first set of experiments provides face and predictive validity for the LHK rat as a model for certain domains of mania. Further investigation including dose-response studies and comparing the efficacy of individual antimanic medications to the efficacy of combined lithium and VPA together are warranted.

The LHK model differs from currently existing models of mania such as the psychomotor stimulant-induced hyperactivity model (Berggren et al. 1978; Furukawa et al. 1975; Jacobs and Silverstone 1986), dopamine transporter knockdown mice (Perry et al*.*^2009^), CLOCK mutant mice (Roybal et al. 2007), or the Madison mice (Scotti et al. 2011) in three aspects. First, it is the first model to induce manic-like behavior through targeting a specific brain region. Second, the induction of an episode of manic-like behavior by kindling (rather than by continuous stimulation) builds on the seminal work of Post and Weiss (1989) of amygdala kindling. However, in their initial work, it was clear that amygdala-kindled rats did not exhibit manic-like behaviors. The third difference between other models and the LHK model is the ability to induce a discrete episode of manic-like behavior during kindling. This brings us closer to the episodic nature of the illness in humans and differs from the life-long manic excitement observed in some other models (Roybal et al. 2007; Scotti et al. 2011). Further studies with longer observation periods are necessary to assess whether the LHK rat cycles from the manic pole of the illness to normality or dips into depressive-like phenotype.

Our data demonstrated that the induction of manic-like behaviors is specific to kindling of the LHT and not other control regions. Several brain imaging (Bhadoria et al. 2003; Cousins et al. 2010; Pearlson et al. 1997; Strakowski et al. 1993) and postmortem studies (Bielau et al. 2005; Brisch et al. 2011) reported hypothalamic abnormalities in patients with bipolar disorder; however, our model provides compelling evidence for the central role the lateral hypothalamus plays in generating certain manifestations of mania. However, it is likely that the elicited behavioral phenotype is not stemming from the LHT solely but rather due to activation of several interfacing neural networks. Mapping cerebral blood flow changes during specific behaviors in freely moving rats using autoradiobiographic techniques (Holschneider and Maarek 2004) is necessary to explore this relevant point.

Kindling has historically been defined in reference to a seizure activity. However, in our study, despite that electrophysiological recordings for seizure activity were not performed, we observed the manic-like behaviors at progressively lower stimulation voltage and earlier during subsequent days of stimulation compared to the first day of stimulation. In addition, we observed the same behaviors during the post-kindling interval which argues that the stimulated neurons were spontaneously firing after the end of direct stimulation which is typical of kindling. Along the same lines, it is important to note that hypothalamic deep brain stimulation has been employed clinically to treat refractory cluster headache (Franzini et al. 2007), self-aggressive behavior in a patient with mental retardation (Hernando et al*.*^2008^), traumatic brain injury (Kuhn et al. 2008), and selected cases of intractable obesity (Whiting et al. 2013) without inducing mania or other psychiatric side effects.

In the context of the current results, it is important to note that the kindling parameters were chosen based on the clinical case report of voltage-dependent mania (Chopra et al*.*^2011^). We only varied stimulation amplitude and fixed frequency, pulse width, and duration of stimulation train. Clinical data from subthalamic nucleus deep brain stimulation for Parkinson's disease show that motor outcome is best modulated by changes in amplitude (Kuncel and Grill 2004; (Balaz et al. 2013). Yet, it is quite possible that different behaviors will be elicited by employing other parameters.

## Conclusion

In conclusion, the spectrum of induced behavioral manifestations combined with the response to antimanic medications proves the LHK rat a novel and valid model for mania.

## Additional files

Additional file 1: Video S1. Sexual behavior, the number of any oral contact with the genital area (erected or flaccid penis or testicles) or immediate perigenital area. Additional file 2: Video S2. Rearing behavior, the number of episodes of standing on the two hind limbs for more than 5-s duration. Additional file 3: Video S3. Grooming behavior, the total time (in 5-s increments) of repeated mouthing of any body part except the genital and perigenital areas or repeated movement of the fore or hind paws over the snout, face, head, trunk, or tail. Additional file 4: Video S4. Feeding behavior: the number of times where the animal is observed holding and chewing food billets; chewing without food or chewing bedding was not considered feeding behavior. Additional file 5: Figure S1. Sexual behavior was observed exclusively in animals that underwent LHK **(A)**, while the total time spent in grooming behavior **(B)** was significantly longer in the LHK compared to NAC and ILC groups during kindling (**P* = 0.009); the frequency of rearing behavior **(C)** was observed significantly more in the LHK compared to NAC and ILC groups during kindling (**P* = 0.0001), and the frequency of feeding behavior **(D)** was also significantly more in the LHK group compared to the NAC and ILC groups during kindling (**P* = 0.0002) by two-way ANOVA; *P* < 0.05 by *post hoc* test; *n* = 7 to 8 per group. Data are expressed as mean ± SEM. Additional file 6: Figure S2. Shows no significant differences in **(A)** the frequency of sexual behavior, **(B)** the duration of grooming behavior, **(C)** the frequency of rearing behavior, or **(D)** the frequency of feeding behavior between voluntary ethanol drinking and water-only drinking rats (*P* > 0.05) by two-way ANOVA; *n* = 8 per group. Data are expressed as mean ± SEM. Additional file 7: Figure S3.
**(A)** shows no significant differences in weight gain or food consumption **(B)** between groups, but lithium-treated rats consumed significantly more water **(C)** compared to saline or VPA-treated rats (*P* < 0.01) by two-way ANOVA; *P* < 0.05 by *post hoc* test; *n* = 8 to 12 per group. Data are expressed as mean ± SEM.
