# Supplementary figures and images for: Lateral hypothalamic kindling induces manic-like behavior in rats: a novel animal model
Source: Int J Bipolar Disord. 2014 Jun 14;2:7. doi: 10.1186/s40345-014-0007-8 (PMC4452639; doi:10.1186/s40345-014-0007-8)

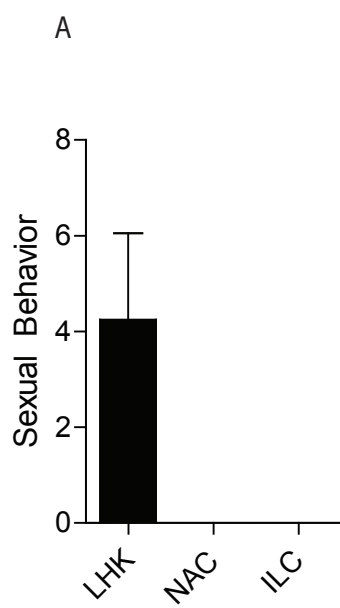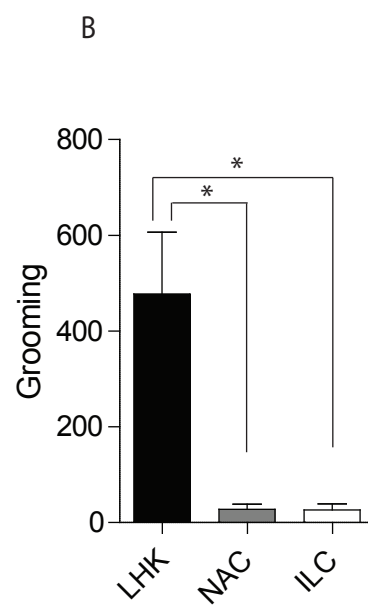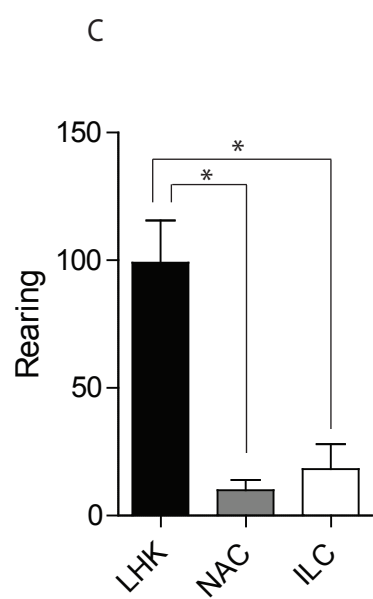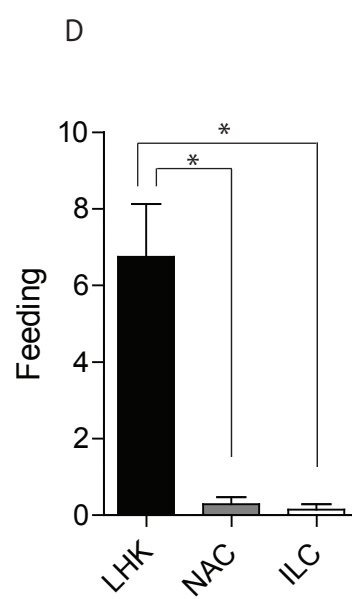

Abulseoud et al ., Supplementary Fig. 1

Supplement: Additional file 5: Figure S1. — Sexual behavior was observed exclusively in animals that underwent LHK (A), while the total time spent in grooming behavior (B) was significantly longer in the LHK compared to NAC and ILC groups during kindling (*P = 0.009); the frequency of rearing behavior (C) was observed significantly more in the LHK compared to NAC and ILC groups during kindling (*P = 0.0001), and the frequency of feeding behavior (D) was also significantly more in the LHK group compared to the NAC and ILC groups during kindling (*P = 0.0002) by two-way ANOVA; P < 0.05 by post hoc test; n = 7 to 8 per group. Data are expressed as mean ± SEM. [file 40345_2014_7_MOESM5_ESM.pdf]

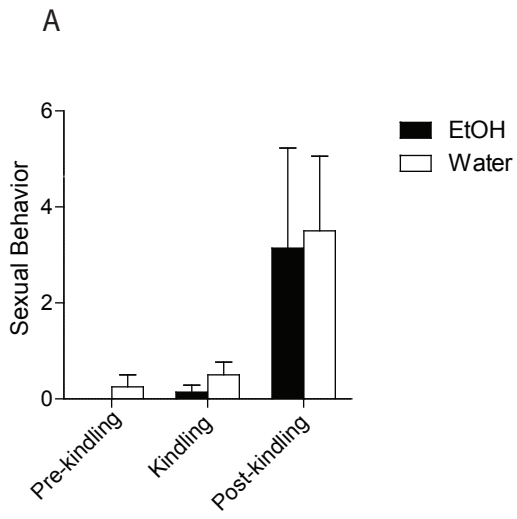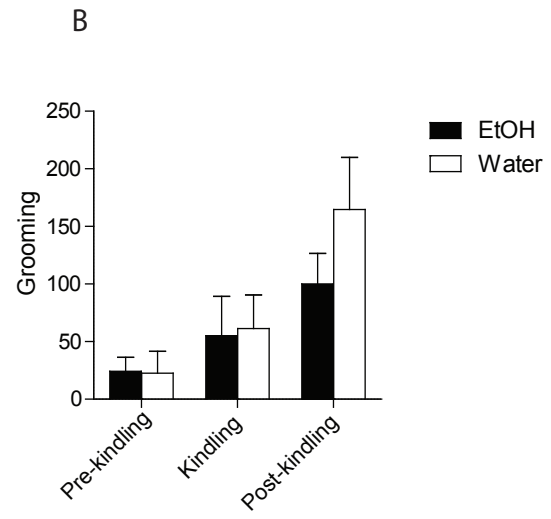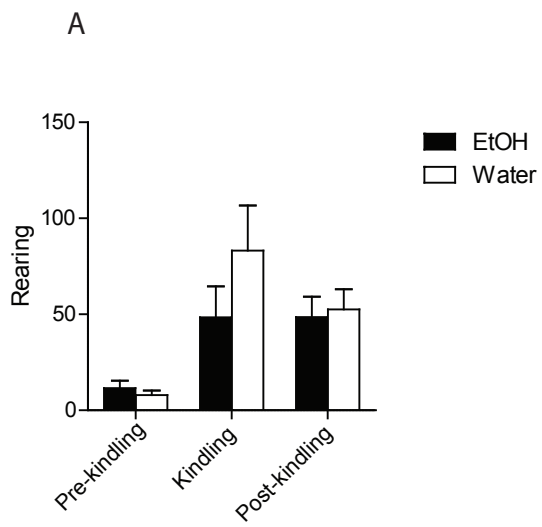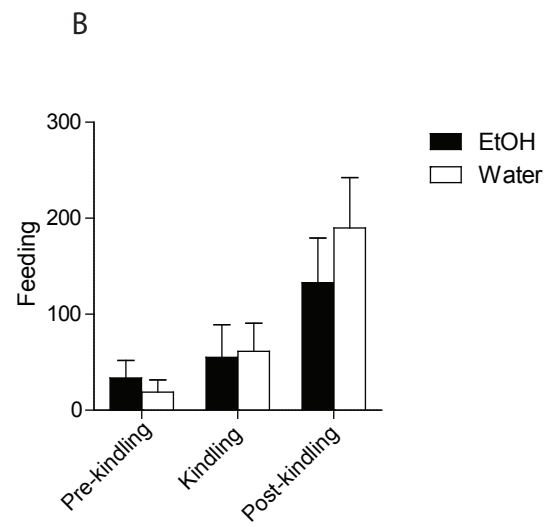

Abulseoud et al ., Supplementary Fig. 2

Supplement: Additional file 6: Figure S2. — Shows no significant differences in (A) the frequency of sexual behavior, (B) the duration of grooming behavior, (C) the frequency of rearing behavior, or (D) the frequency of feeding behavior between voluntary ethanol drinking and water-only drinking rats (P > 0.05) by two-way ANOVA; n = 8 per group. Data are expressed as mean ± SEM. [file 40345_2014_7_MOESM6_ESM.pdf]

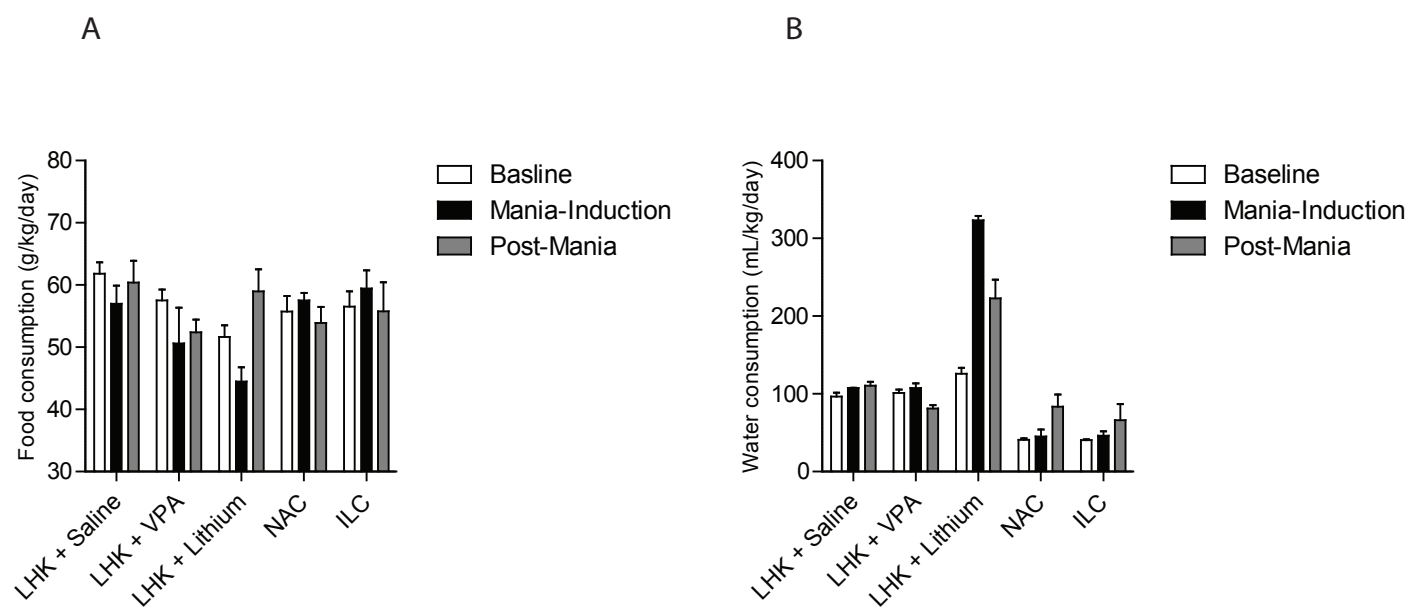

Supplement: Additional file 7: Figure S3. — (A) shows no significant differences in weight gain or food consumption (B) between groups, but lithium-treated rats consumed significantly more water (C) compared to saline or VPA-treated rats (P < 0.01) by two-way ANOVA; P < 0.05 by post hoc test; n = 8 to 12 per group. Data are expressed as mean ± SEM. [file 40345_2014_7_MOESM7_ESM.pdf]
